# Psychometric properties and cultural adaptation of the Polish Version of the Gay Affirmative Practice Scale

**DOI:** 10.3389/fpubh.2024.1384429

**Published:** 2024-05-02

**Authors:** Piotr Karniej, Anthony Dissen, Raúl Juárez-Vela, Iván Santolalla-Arnedo, Teresa Sufrate-Sorzano, Maria Elena Garrote-Camara, Michał Czapla

**Affiliations:** ^1^Faculty of Economics, WSB MERITO University in Wroclaw, Wrocław, Poland; ^2^Group of Research in Care (GRUPAC), Faculty of Health Sciences, University of La Rioja, Logroño, Spain; ^3^School of Health Sciences, Stockton University, Galloway, NJ, United States; ^4^Department of Emergency Medical Service, Wrocław Medical University, Wrocław, Poland; ^5^Institute of Heart Diseases, University Hospital, Wrocław, Poland

**Keywords:** gender minorities, gay people, gay affirmative, acceptability of healthcare, gay, LGBT healthcare

## Abstract

**Introduction:**

The lesbian, gay, bisexual, and transgender (LGBT) people often face unique medical disparities, including obstacles to accessing adequate and respectful care. The purpose of this study was to test the psychometric properties(internal consistency, reliability, and factor structure) of the Polish-language version of the Gay Affirmative Practice Scale (GAP-PL).

**Material:**

The study was conducted over a 6-month period in 2023, from February to June, involving 329 medical students and professionals who evaluated the GAP-PL.

**Methods:**

Before testing the psychometric properties of the original Gay Affirmative Practice Scale (GAP), it was translated and adapted from the original English language version into the Polish language. Authors then tested the psychometric properties of the tool on a sample of 329 participants. The internal coherence of the questionnaire was tested with the analysis of verifying factors (Confirmatory Factor Analysis). Cronbach alpha and the discriminatory power index were used as internal consistency measures.

**Results:**

There were more female than male participants (55.32%). More than 53% of the participants were heterosexual, and the average age of the respondents was ~30 years. The internal consistency of the Polish-language version and its domains was strong with the overall Cronbach's alpha ranges for each subscale domains ranging between 0.936 and 0.949. The McDonald's omega coefficient was 0.963.

**Conclusion:**

The GAP-PL has excellent properties of factorial validity and can be used in research and clinical practice in Polish-speaking populations.

## 1 Introduction

The lesbian, gay, bisexual, and transgender (LGBT) community is characterized by significant racial, ethnic, and socioeconomic diversity, yet collectively faces a barrier to accessing appropriate health care (1). The challenges they face include not only issues of access to health services and culturally competent care, but also the impact of national policies that often exacerbate social stigma, marginalization, or discrimination (2). Recent years have seen advances in the accurate collection of data, research, and a deeper understanding of the healthcare needs of the LGBT community and the challenges related to access to care (3–5). Although significant steps have been taken to reduce health disparities among LGBT individuals, further action is needed to achieve full equality in the healthcare system, especially in Poland. Poland currently has a high rate of discrimination based on sexual orientation (6), and both conscious and unconscious homophobia in society often stem from the activities of right-wing politicians, for whom the LGBT community is a convenient target for criticizing nationalist values (7).

The existence of non-heteronormative individuals in Polish society has been and is evident, but for years this topic has been treated as taboo and not discussed in family or public relations (8). The discourse constructing homosexuality as a threat to the nation has been used for many years by both general members of Polish society and politicians in Poland to legitimize homophobic rhetoric and behaviors, causing prejudices against LGBT people at the level of individual citizens' beliefs (9). Political and partly social opposition to the rights of sexual minorities in Poland is among the highest in the EU. In the research conducted by ILGA-Europe in 2023 (10), 2 Poland scored only 15% in respecting human rights in the area of equality, ranking close to Belarus (12%) and Russia (8%). Countries with the highest rates in Europe have been for many years Malta (89%), Denmark (76%), and Spain (74%) (10).

For these reasons, quality measurement of medical care in the context of affirming practices toward LGBT individuals has not been conducted in Poland, which seems necessary given the evidence demonstrated in the literature. Given such an unfavorable environment for the LGBT community in Poland, and simultaneously recognizing that the healthcare needs of LGBT individuals are often different from those of the heterosexual majority (11), there may be exclusion of gays, lesbians, bisexuals, and transgender individuals in terms of access to proper medical care, particularly in the sense of health services that recognize the patient as a non-heteronormative individual. In the latest report by the KPH (Campaign Against Homophobia) organization on the situation of LGBT people in Poland, it was shown that when sexual orientation was known to healthcare workers, they were provided with poorer quality care and received overall worse treatment, with this discriminatory behavior most often being directed toward transgender individuals (48%). Surveyed individuals described incidents such as refusal to perform a procedure, inappropriate jokes, or entering information about sexual orientation into medical documentation (12).

An important phenomenon in the experience of treating people from the LGBT community in health care is minority stress (13), which has been widely described in the literature, including the identification of the impact of this stress on health, mainly in mental and emotional disorders, with minority stress being seen and treated as a stressor in this case (14). One type of proximal stressor directly related to the person providing the health service may be the attitude of a doctor, nurse, or other medical professional, whose affirmative or stigmatizing attitude may translate not only into the experience of the health service itself, but also into the patient's eventual refusal to use the service, which is known as the self-exclusion phenomenon. Minority stressors therefore have a unique impact on health as they add up to the effect of general stressors (15). A study of minority stress levels of the LGBT community conducted in Poland showed that minority stress is not only noticeably present, but is significantly higher than stress due to other health determinants (15).

This collectively highlights the reasons why it is important to assess and improve the clinical skills, attitudes, beliefs, and cultural understanding of healthcare providers, especially medical doctors, nurses, paramedics, and other allied healthcare professionals. This is essential to enable them to effectively meet the diverse needs of LGBT patients, and not further contribute to feelings of stigma, shame, and other health disparities that result from a lack of competence (16).

Various scales are available worldwide to measure acceptance or affirmation toward the LGBTQ community, including those focusing on people living with HIV, gay men, lesbians, same-sex families, and individuals receiving pre-exposure prophylaxis (PrEP) (17–20). In the context of the Health Exclusion Research in Europe (HERE) project, Poland has made strides by developing a tool focused on enhancing clinical skills in LGBT care (21). Despite this progress, there is still a notable lack of instruments assessing affirmation toward homosexual individuals specifically. This gap highlights the need for new methodologies that allow a more comprehensive and inclusive approach within the Polish healthcare system. In choosing the GAP questionnaire for our study, we aimed to address this gap.

Therefore, the purpose of this study is to evaluate the psychometric properties (internal consistency, reliability, and factor structure) of the Polish-language version of the Gay Affirmative Practice Scale (GAP-PL).

## 2 Materials and methods

### 2.1 Study population

A sample of 329 study participants took part in the testing of the GAP-PL, which took place during the first 6-months of 2023 (February to June). According to Parra, 5–10 participants per item should be recruited for sample calculations (22). As such, the minimum sample size should be 150 participants for the 30-item GAP-PL questionnaire. All participants were fully informed of the purposes of the study and gave their consent prior to study participation, after which participants were asked to complete an anonymous, two-phase questionnaire. Inclusion criteria was for participants to have an active status as a medical student or a medical professional. Phase 1 of the questionnaire asked participants to share demographics (i.e., age, gender identity, place of residence, sexual orientation, and medical profession). Phase 2 of the questionnaire contained the finalized text of the questions for the GAP-PL. For the purpose of ensuring data integrity and preventing multiple submissions from the same respondents, our study utilized an automated IP filtering feature provided by the web platform webankieta (23). It's important to note that this process was entirely automated and did not involve any direct access to individual IP addresses by the researchers. Furthermore, no instances of repeated survey submissions were detected, and the IP addresses were not analyzed or used for any purpose beyond maintaining data quality and integrity. This approach aligns with strict data privacy standards, ensuring that IP addresses were used solely for these specific technical purposes without compromising respondent anonymity.

### 2.2 The Gay Affirmative Practice Scale

The Gay Affirmative Practice (GAP) Scale is an assessment tool designed by Catherine Crisp to measure clinicians' beliefs and behaviors in relation to the treatment of LGTB clients (24). It contains statements with which respondents may agree or disagree, thus assessing their attitudes and practices in working with LGBT clients. The GAP Scale focuses on promoting affirmative practice and attitudes toward LGBT people, encouraging medical professionals to educate, respect, and support this community. It is a scale constructed from 30 questions in two domains (Beliefs and Behaviors). The first domain (questions 1–15) requires responses defined by a Likert scale (strongly agree, agree, neither agree nor disagree, disagree, strongly disagree). Examples of questions from the first domain are: “Practitioners should educate themselves about gay/lesbian lifestyles,” “Practitioners should take advantage of professional development opportunities to improve their practice with gay/lesbian clients.” The second domain of the questionnaire (questions 16–30) requires answers: always, usually, sometimes, rarely, never. The answers given are assigned a certain number of points from 1 to 5, according to a table defined in the scale. Examples of questions from the second domain are: “I inform clients about gay affirmative resources in the community,” “I show comfort with gay/lesbian issues to gay/lesbian clients.” A higher score indicates a more affirmative practice toward LGBT clients. In the original GAP Scale, high Cronbach's alpha values were demonstrated, indicating its reliability and internal consistency. The Cronbach's alpha was 0.93 for the belief domain and 0.94 for the behavior domain in the original English-language version (24).

### 2.3 Instrument translation and cultural and language adaptation

In using the questionnaire for its adaptation into Polish, researchers obtained permission from the author of the original instrument, C. Crisp, for such an adaptation. In order to properly translate the GAP Scale into Polish, researchers followed the guidelines and process for translation as described by Beaton et al. (25). These steps (translation, synthesis, back translation, synthesis of back translation, expert committee review of the transcribed version, and pre-testing) were carried out throughout the process of developing the Polish language version of the GAP Scale to ensure that the translation was correct both linguistically and culturally. Two independent qualifies translators were involved in the initial translation of the English version of the GAP Scale into Polish. Next, the initial Polish language version was translated back into English in a blinded process to ensure that the English-speaking bilingual translators had no prior experience working with the original version of the instrument. For the next phase of this process, an English speaker subsequently checked the back-translated instrument to ensure that the questions and prompt scenarios matched the original meaning, purpose, and language of the GAP Scale prior to translation. The document was then reviewed by an expert committee which consisted of: a health promotion specialist, a public health specialist, a paramedic, dietitians, a nurse, a psychologist, and a physician. At the end of the phase, the cognitive interviews were conducted with 50 volunteers. Edits and changes were made until final agreement was reached regarding the Polish-language (GAP-PL) version of the instrument (see Supplementary material).

### 2.4 Ethical review and approval

All procedures and study methods were reviewed by the Bioethics Committee of Wrocław Medical University in Poland prior to study implementation (No. KB 976/2022). As part of the informed consent procedures for this study, all subjects were informed that their participation was purely voluntary, all data would remain anonymous, no data would be collected that contained personally identifying information, and that subjects could end their participation at any time. This information would summarily provided to participants in written format.

### 2.5 Statistical analysis

Internal consistency of the questionnaire was checked with Confirmatory Factor Analysis (CFA). Standardized Root Mean Square Residual (SRMR), Root Mean Square Error of Approximation (RMSEA), Comparative Fit Index (CFI), and Tucker-Lewis Index (TLI) were used within the Hu-Bentler two-index strategy to assess CFA's goodness of fit (SRMR <0.09 plus additionally one of the conditions CFI > 0.96, TLI > 0.96 or RMSEA <0.06). As the GAP Scale items are expressed on an ordinal rather than a continuous scale, the Diagonally Weighted Least Squares method was used. Cronbach's alpha (α) together with discriminative power index were used as internal consistency measures. The following thresholds for internal consistency were used: 0.9 ≤ α—excellent; 0.8 ≤ α <0.9—good; 0.7 ≤ α <0.8—acceptable; 0.6 ≤ α <0.7—questionable; 0.5 ≤ α <0.6—poor; and α <0.5—unacceptable. R 4.2.2 was used along with RStudio GUI and psy, lavaan, psych, and diagram packages (26–31).

## 3 Results

### 3.1 Group characteristic

The characteristics of the study group are shown in Table 1. Over 55% of participants identified as female. More than 56% of the participants identified their orientation as heterosexual. The mean age was 30.9 years. The largest proportions of participants were doctors (26.44%), nurses (22.49%), and dietitians (20.97%). Almost 82% of participants had never received any training in working with LGBT patients.

**Table 1:** Characteristics of the study group.

**Parameter**		**Total (*N* = 329)**
Gender	Female	182 (55.32%)
	Male	144 (43.77%)
	Non-binary person	1 (0.30%)
	Other	2 (0.61%)
Age [years]	Mean (SD)	30.89 (8.82)
	Median (quartiles)	30 (25–36)
	Range	18–63
	*N*	329
Place of residence	Rural area	44 (13.37%)
	City <20th. inhab.	18 (5.47%)
	City 20–100th. inhab.	46 (13.98%)
	City 100–500th. inhab.	47 (14.29%)
	City >500th. inhab.	174 (52.89%)
Sexual orientation	Heterosexual	186 (56.53%)
	Homosexual	105 (31.91%)
	Bisexual	34 (10.33%)
	Other	2 (0.61%)
	I prefer not to answer	2 (0.61%)
Relationship status	In an informal relationship	145 (44.07%)
	Single	104 (31.61%)
	Married to a man	48 (14.59%)
	Married to a woman	18 (5.47%)
	Divorced	7 (2.13%)
	In an informal relationship (e.g., concluded abroad)	6 (1.82%)
	Widowed	1 (0.30%)
Profession	Physician	87 (26.44%)
	Student	74 (22.49%)
	Nurse	69 (20.97%)
	Dietitian	44 (13.37%)
	Paramedic	19 (5.78%)
	Physiotherapist	13 (3.95%)
	Dentist	8 (2.43%)
	Midwife	5 (1.52%)
	Pharmacist	4 (1.22%)
	Laboratory diagnostician	1 (0.30%)
	Other	5 (1.52%)
How many trainings (workshops and webinars) have you attended in the last 5 years that dealt in any way with LGBT patient issues?	None	267 (81.16%)
	1–2 times	46 (13.98%)
	3–5 times	10 (3.04%)
	Over 5 times	6 (1.82%)

The GAP Scale questionnaire assesses respondents' beliefs about their approach to and their own behavior toward LGBT patients/clients. The score on each scale is a number between 15 and 75 points. The higher the score, the more affirmative the attitude. For the GAP Scale, no norms were set for how many points earned can be considered affirmative. The mean score obtained by respondents on the belief scale was 66.14 points (SD = 9.46). The mean score obtained by respondents on the behavior scale was 54.37 points (SD = 14.83), see Table 2.

**Table 2:** Results for each subscale.

**GAP**	**Point range**	***N***	**Mean**	**SD**	**Median**	**Min**	**Max**	**Q1**	**Q3**
Beliefs	15–75	329	66.14	9.46	68	15	75	61	74
Behaviors	15–75	329	54.37	14.83	57	15	75	47	65

### 3.2 Analysis of the individual questionnaire items

Table 3 shows the results of the analysis of the individual questionnaire items. There was a high ceiling effect in questions 2.

**Table 3:** Analysis of the individual questionnaire items.

**Item**	**Floor effect**	**Ceiling effect**
1	2.1%	60.2%
2	0.6%	84.5%
3	1.5%	60.5%
4	1.2%	62.6%
5	2.1%	44.1%
6	1.2%	54.4%
7	1.2%	71.1%
8	0.9%	59.9%
9	1.8%	36.8%
10	0.9%	54.4%
11	0.9%	61.7%
12	0.9%	58.1%
13	0.9%	57.4%
14	0.9%	61.7%
15	1.2%	62.3%
16	11.9%	41.9%
17	14.6%	35.3%
18	28.6%	18.2%
19	11.9%	35.3%
20	12.2%	42.2%
21	23.4%	28.9%
22	33.1%	22.8%
23	9.1%	66.3%
24	7.9%	53.5%
25	28.9%	15.2%
26	13.4%	20.7%
27	6.7%	66.3%
28	9.7%	53.8%
29	8.2%	63.8%
30	15.2%	35.3%

### 3.3 Confirmatory factor analysis

The original GAP structure is 2-factor: beliefs (items no. 1–15) and behaviors (items no. 16–30). Satisfactory fit indices were obtained for this structure (see Table 4).

**Table 4:** Results of fit indices.

**Test chi-kwadrat**			**RMSEA**	**CFI**	**TLI**	**SRMR**
χ^2^	**Df**	***P***				
337,647	404	0,993	<0,001	>0,999	>0,999	0,066

### 3.4 Internal consistency analysis of the GAP scale

**Figure 1:**
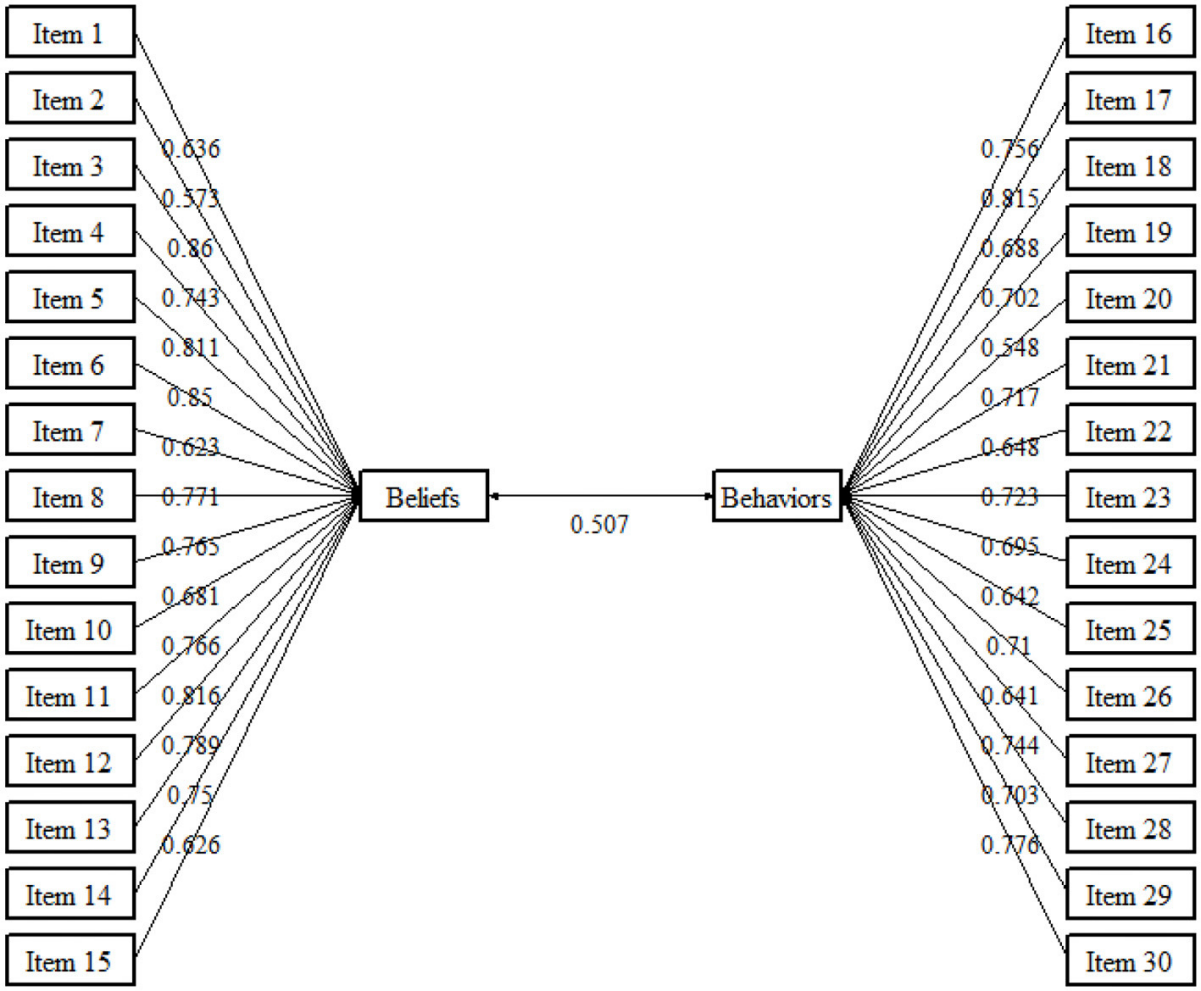
Path diagram for CFA of the Polish Version of the GAP Scale.

Table 5 shows the analysis of the internal consistency of the GAP-PL. The loadings of individual items ranged from 0.548 to 0.86 and were statistically significant (*p* < 0.05). Cronbach's alpha was excellent and for each of the subscale domains ranged between 0.936 and 0.949. Also, the McDonald's omega index (ω)-value indicates the high reliability of the scale, which was 0.963. Figure 1 shows the path diagram for CFA of the GAP-PL.

**Table 5:** The analysis of the internal consistency of the GAP-PL.

**Domain**	**Item**	**Loading**	***p***	**Cronbach's alpha**
Beliefs	1	0.636	*p* < 0.001	
	2	0.573	*p* < 0.001	
	3	0.860	*p* < 0.001	
	4	0.743	*p* < 0.001	
	5	0.811	*p* < 0.001	
	6	0.850	*p* < 0.001	
	7	0.623	*p* < 0.001	0.949
	8	0.771	*p* < 0.001	
	9	0.765	*p* < 0.001	
	10	0.681	*p* < 0.001	
	11	0.766	*p* < 0.001	
	12	0.816	*p* < 0.001	
	13	0.789	*p* < 0.001	
	14	0.750	*p* < 0.001	
	15	0.626	*p* < 0.001	
Behaviors	16	0.756	*p* < 0.001	
	17	0.815	*p* < 0.001	
	18	0.688	*p* < 0.001	
	19	0.702	*p* < 0.001	
	20	0.548	*p* < 0.001	
	21	0.717	*p* < 0.001	
	22	0.648	*p* < 0.001	
	23	0.723	*p* < 0.001	0.936
	24	0.695	*p* < 0.001	
	25	0.642	*p* < 0.001	
	26	0.710	*p* < 0.001	
	27	0.641	*p* < 0.001	
	28	0.744	*p* < 0.001	
	29	0.703	*p* < 0.001	
	30	0.776	*p* < 0.001	

### 3.5 Cronbach's alpha after excluding individual items

Cronbach's Alpha stands at 0.949 and 0.936 in the Beliefs section and the Behaviors section, respectively. Analysis indicates that excluding any single item from these sections does not significantly increase Cronbach's Alpha. This suggests that both scales are robustly constructed, as demonstrated by the high alpha values (Table 6).

**Table 6:** Cronbach's alpha after excluding individual items.

	**Item**	**Cronbach's alpha if item deleted**
Beliefs	1	0.950a
	2	0.947
	3	0.945
	4	0.945
	5	0.946
	6	0.945
	7	0.947
	8	0.944
	9	0.947
	10	0.946
	11	0.944
	12	0.944
	13	0.945
	14	0.946
	15	0.947
Behaviors	16	0.930
	17	0.929
	18	0.933
	19	0.932
	20	0.935
	21	0.931
	22	0.933
	23	0.932
	24	0.931
	25	0.933
	26	0.933
	27	0.934
	28	0.930
	29	0.930
	30	0.931

## 4 Discussion

The specific psychometric properties examined in the study of the Polish-language version of the Gay Affirmative Practice Scale (GAP-PL) were internal consistency, reliability, and factor structure. Results indicate that the psychometric properties and cultural adaptation of the GAP-PL were carried out successfully. This is the first study adapting the GAP Scale into the Polish language in order to improve our understanding of the dimensions measured by the GAP (Beliefs and Behaviors). This achievement enables the creation of an assessment scale that is culturally equivalent for evaluating clinical skills in the context of Polish-speaking healthcare professionals. The process of cross-cultural adaptation went smoothly, with no language challenges, and only a few expressions were adjusted slightly to ensure their cultural relevance. The internal consistency of the GAP Scale of each of the two domains contained within the assessment scale were high, with values very close to those found in the original version of the GAP Scale. The Cronbach's alpha for the two subscales of the GAP-PL was 0.95 for the belief domain and 0.94 for the behavior domain. Similar alpha values were reported by the Crips of the original questionnaire, with 0.93 for the belief domain and 0.94 for the behavior domain (24).

The scale has important implications for patient care practice, education, and health care research. By identifying areas of competency deficits or inappropriate behavior toward LGBT patients, it helps to develop the awareness and skills needed to effectively support LGBT identifying clients. The GAP-PL is an example of a scale that contributes to promoting equality and understanding of LGBT people that can be used in the fields of public health and clinical medical care. Developed by C. Crisp in 2006, the Gay Affirmative Practice Scale (GAP) is established in the field LGBT health research; however, its adaptation into the Polish language represents a new application, as it has not been previously tailored for use in the Polish language despite a substantial body of literature that underscores its effectiveness and importance (24).

In the literature, the GAP Scale is used both to engage social work therapists in gay and lesbian affirmative practice (32), determining health professionals' attitudes toward gay and lesbian, bisexual and transgender people seeking health care for their children in family-centered pediatric hospitals (33), or as a scale to support psychotherapeutic work in identifying factors that may influence the extent to which LGBT affirmative practice would be positively related to psychological wellbeing and assessing levels of internalized homophobia (34). The aspect of medical professionals' attitudes toward affirmative care for children from LGBT backgrounds is not isolated as it is also receiving attention at the level of academic nursing education (24). All of these studies used the GAP Scale questionnaire and found it to be of high research quality.

Numerous examples of the application of the GAP questionnaire can be found in the literature, not only for social workers, psychologists, and students, but also for practicing nurses (35). In this study, the authors point out that although Italian nurses showed moderately positive attitudes and affirmative behavior, there is a need to increase their cultural competence in relation to sexual minorities.

Authors using the GAP Scale questionnaire in a number of countries around the world argue that increasing cultural competence includes, among other things, using inclusive language, increasing knowledge about sexual minorities, using educational resources such as cultural competency workshops, experiential stories, and seeking mentoring from people with experience of working with sexual minorities (36). The GAP Scale is also applicable to assess the attitudes, beliefs, and intentions of medical and social professionals toward older people from the LGBT community. Indeed, lifelong stigma can affect barriers to care, social isolation, and accompanying health disparities (37). As the literature shows, the GAP Scale, despite its short history, is a widely used research scale with proven effectiveness in assessing the affirmative competence of health professionals. Therefore, there is scientific evidence for its use also in Polish speaking communities and populations to assess the competence of service providers. Although our study was conducted on a group of health professionals and medical students (which is in line with previous use of the GAP Scale in other publications), further research could be extended to include social service professionals, as encouraged by other publications.

### 4.1 Study limitation

Important limitations should be acknowledged in this study. Although the sample size was adequate for addressing the primary research goals, a more comprehensive viewpoint could be achieved by increasing the number of participants. Additionally, it's important to highlight that our study exclusively utilized an online platform for data gathering, which could have excluded certain potential participants, particularly those who lacked internet access.

## 5 Conclusion

The results of this study of translation and cultural adaptation the Polish Version of the Gay Affirmative Practice Scale (GAP-PL) show that the internal consistency, reliability, and factor structure of the scale are excellent. The GAP-PL will be useful, inter alia, for research purposes in the healthcare field. Compared to the original instrument, the psychometric tests and the results of the transcultural adaptation were similar to the original English-language validated version of the GAP Scale.

## Data availability statement

The raw data supporting the conclusions of this article will be made available by the authors, without undue reservation.

## Ethics statement

The studies involving humans were approved by Bioethics Committee of Wrocław Medical University in Poland (No. KB 976/2022). The studies were conducted in accordance with the local legislation and institutional requirements. Written informed consent for participation was not required from the participants or the participants' legal guardians/next of kin in accordance with the national legislation and institutional requirements.

## Author contributions

PK: Conceptualization, Data curation, Formal analysis, Funding acquisition, Investigation, Methodology, Project administration, Resources, Software, Validation, Visualization, Writing – original draft, Writing – review & editing. AD: Conceptualization, Formal analysis, Writing – original draft, Writing – review & editing. RJ-V: Formal analysis, Writing – original draft, Writing – review & editing. IS-A: Writing – original draft, Writing – review & editing. TS-S: Writing – original draft, Writing – review & editing. MG-C: Writing – original draft, Writing – review & editing. MC: Conceptualization, Formal analysis, Supervision, Writing – original draft, Writing – review & editing.
