# Supplementary material for: Psychometric properties and cultural adaptation of the Polish Version of the Gay Affirmative Practice Scale
Source: Front Public Health. 2024 May 2;12:1384429. doi: 10.3389/fpubh.2024.1384429 (PMC11097662; doi:10.3389/fpubh.2024.1384429)
Supplement: Supplementary file 1 [file Data_Sheet_1.doc]

**Skala Praktyk Afirmatywnych Wobec Osób Homoseksualnych
Gay Affirmative Practice Scale PL(GAP-PL)**

Ten kwestionariusz ma na celu zmierzenie przekonań pracowników ochrony zdrowia na temat leczenia pacjentów homoseksualnych oraz ich zachowań w środowisku klinicznym z tymi pacjentami. Nie ma dobrych ani złych odpowiedzi. Proszę odpowiadać na każde pytanie jak najszczerzej.

Proszę ocenić, w jakim stopniu zgadzasz się lub nie zgadzasz się z każdym stwierdzeniem dotyczącym postępowania z pacjentem homoseksualnym na podstawie poniższej skali:

ZZ = zdecydowanie się zgadzam
Z = zgadzam się
N = Ani się zgadzam, ani się nie zgadzam
NZ = Nie zgadzam się
ZNZ = Zdecydowanie się nie zgadzam

1. W swojej praktyce z pacjentami homoseksualnymi, profesjonaliści medyczni powinni wspierać zróżnicowany skład ich rodzin. _______
2. Profesjonaliści medyczni powinni werbalizować szacunek dla stylu życia pacjentów _______
   homoseksualnych.
3. Profesjonaliści medyczni powinni dołożyć starań, aby poznać różnorodność _______
   pośród społeczności homoseksualnej.
4. Profesjonaliści medyczni powinni posiadać wiedzę na temat zasobów z których mogą _______
   korzystać osoby homoseksualne (np. centra szkoleniowe, organizacje, centra wsparcia,
   centra zdrowia seksualnego)
5. Profesjonaliści medyczni powinni edukować się na temat stylu życia gejów i lesbijek. _______
6. Profesjonaliści medyczni powinni pomagać pacjentom homoseksualnym rozwijać _______
   pozytywną tożsamość jako osoby homoseksualne.
7. Profesjonaliści medyczni powinni kwestionować błędne informacje na temat pacjentów _______ homoseksualnych.
8. Profesjonaliści medyczni powinni pracować nad rozwijaniem umiejętności niezbędnych do efektywnej pracy z pacjentami będącymi osobami homoseksualnymi _______
9. Profesjonaliści medyczni powinni zachęcać pacjentów homoseksualnych do tworzenia _______
   sieci, które wspierają ich jako osoby homoseksualne.
10. Profesjonaliści medyczni powinni posiadać wiedzę na temat problemów charakterystycznych _______
    dla par homoseksualnych.
11. Profesjonaliści medyczni powinni zdobyć wiedzę niezbędną do efektywnej pracy _______
    z pacjentami homoseksualnymi.
12. Profesjonaliści medyczni powinni pracować nad rozwijaniem umiejętności niezbędnych _______
    do efektywnej pracy z pacjentami będącymi osobami homoseksualnymi.
13. Profesjonaliści medyczni powinni pracować nad rozwojem postaw niezbędnych do _______
    skutecznej praktyki z pacjentami będącymi osobami homoseksualnymi
14. Profesjonaliści medyczni powinni pomagać pacjentom redukować wstyd z powodu uczuć _______ homoseksualnych.
15. Dyskryminacja stwarza problemy, którymi pacjenci będący osobami homoseksualnymi _______
    mogą chcieć zająć się w trakcie leczenia.

Proszę ocenić, jak często angażujesz się w każde z zachowań w stosunku do homoseksualnych pacjentów na podstawie następującej skali:

ZAW = Zawsze
Z = Zwykle
CZAS = Czasami
R = Rzadko
N = Nigdy

1. Pomagam pacjentom redukować wstyd z powodu uczuć homoseksualnych. _______
2. Pomagam pacjentom homoseksualnym zajmować się problemami wynikającymi _______
   z uprzedzeń społecznych.
3. Informuję pacjentów o miejscach, w których osoba homoseksualna może uzyskać wsparcie w zwiększeniu własnej akceptacji. _______
4. Dostrzegam wpływ homofobii na swoich pacjentów. _______
5. Odnoszę się do orientacji seksualnej pacjenta, jeśli jest to istotne dla leczenia. _______
6. Pomagam pacjentom homoseksualnym przezwyciężyć religijną opresję, której doświadczyli _______
   w związku z ich orientacją seksualną.
7. Prowadzę interwencje, które zwiększają bezpieczeństwo pacjentów homoseksualnych. _______
8. Zwracam uwagę, że orientacja homoseksualna jest tak samo zdrowa jak _______
   orientacja heteroseksualna.
9. Czuję się komfortowo rozmawiając o sprawach homoseksualnych z pacjentami _______
   homoseksualnymi
10. Pomagam pacjentom zidentyfikować u nich uwewnętrznioną homofobię. _______
11. Edukuję się na temat problemów osób homoseksualnych. _______
12. Jestem otwarty(a) na osobiste uwarunkowania pacjenta (np. styl życia, obecność partnera(ki), otwartość wobec otoczenia) podczas dostosowywania leczenia pacjentów homoseksualnych. _______
13. Tworzę klimat, który pozwala na dobrowolną samoidentyfikację przez pacjentów homoseksualnych _______
14. O orientacji seksualnej rozmawiam z klientami w sposób niezagrażający. _______
15. Ułatwiam pacjentom homoseksualnym wyrażanie gniewu z powodu opresji, której doświadczyli. _______

**Instrukcje dotyczące punktacji:** Korzystając z poniższej tabeli, każdej odpowiedzi przypisz wskazaną liczbę punktów. Po udzieleniu odpowiedzi na wszystkie pytania należy zsumować otrzymane punkty. Wyższe wyniki odzwierciedlają bardziej afirmatywną praktykę wobec pacjentów homoseksualnych.

| **Pozycje 1–15** | **Pozycje 16–30** | **Punkty** |
| --- | --- | --- |
| Zdecydowanie się zgadzam | Zawsze | **5** |
| Zgadzam się | Zwykle | **4** |
| Ani się zgadzam, ani się nie zgadzam | Czasami | **3** |
| Nie zgadzam się | Rzadko | **2** |
| Zdecydowanie się nie zgadzam | Nigdy | **1** |

Cytowanie: Karniej P, Dissen A, Juarez-Vela R, Santolalla-Arnedo I, Sufrate-Sorzano T, Garrote-Camara ME and Czapla M (2024) Psychometric properties and cultural adaptation of the Polish Version of the Gay Armative Practice Scale. Front. Public Health 12:1384429. doi: 10.3389/fpubh.2024.1384429
